# DCB therapy: quo vadis?

**DOI:** 10.1007/s00392-025-02733-1

**Published:** 2025-08-06

**Authors:** Bruno Scheller, Bernhard Haring

**Affiliations:** 1 Clinical and Experimental Interventional Cardiology, University of Saarland, Homburg/Saar, Germany; 2 Department of Internal Medicine III, Saarland University Hospital, Homburg/Saar, Germany; 3 Department of Medicine IV, Clinic Hietzing, Vienna, Austria

Sirs,

At the 2024 Heart Days meeting of the German Cardiac Society (DGK) in Hamburg, a session of the Interventional Cardiology Working Group (AGIK) entitled ‘DCB—Here to stay?’ was focused on the role of drug coated balloons (DCB) in the treatment of coronary artery disease. It was common sense, that DCB therapy is currently not a valid option given the lack of evidence and actual guidelines. In fact, this critical view on DCB therapy is supported by recent European Society of Cardiology (ESC) guidelines on the management of chronic coronary syndromes (CCS) [1]. A few years ago, DCB therapy had been considered a recommended procedure for the treatment of in-stent restenosis [2], but this has been retracted in the latest CCS guidelines [1]. The reasons for this downgrade have been discussed controversially, especially the focus on efficiency (target lesion revascularization) while not observing hard clinical endpoints such as myocardial infarction and death being in favor of DCB treatment by avoiding additional stent layers [3]. There was also consensus in the ESC guidelines that DCB have no role in the treatment of de novo lesions [2]. But do these views on DCB therapy actually reflect the available clinical evidence [4–8]?

DCB therapy is one of the hot topics at international scientific meetings. The number of scientific publications on DCB therapy is increasing exponentially and so is the evidence for DCB therapy [7, 9]. One may therefore ask: Is the criticism and neglect of DCB therapy still justified?

Importantly, this treatment method is not new. The first experimental description of the procedure was published more than 20 years ago [10], followed by the first-in-man study in the *New England Journal of Medicine* [11]. The justification for DCB are the limitations of drug eluting stents (DES). The rate of percutaneous coronary interventions (PCI) for in-stent restenosis is still up to 10% of all PCI [12]. Beyond the first year after DES implantation, a device-associated event rate of 2 to 3% can be expected without flattening of the curve over time [13, 14]. The risk increases with the number and length of the implanted DES and increases further as the vessel diameter decreases [14]. Patients with risk factors such as diabetes mellitus are particularly affected [14]. It therefore makes sense to consider alternatives to permanent implants, at least to reduce their total number and length [9].

Baumer and colleagues deserve recognition and congratulations for their contribution to the growing clinical evidence of DCB therapy [15]. In the current issue of *Clinical Research in Cardiology*, they present a propensity score matched analysis of 606 patients with treatment of de novo lesions with either DES or DCB. The median follow-up time was 5.7 years without significant differences in cardiovascular or all-cause mortality, major adverse cardiovascular events (MACE), acute myocardial infarction, or any revascularization between DES and DCB. Importantly, the authors observed a trend toward lower rates of target lesion revascularization in patients with small vessel disease (SVD), and in bifurcation side branch lesions in favor of DCB treatment [15].

Their findings compare well to randomized clinical trials (RCT) in SVD. The largest RCT to date, the Basket Small 2 study showed non-inferiority of ‘DCB only’ therapy to DES up to three years [4, 5]. The REC-CAGEFREE I study including non-complex coronary artery disease, which is frequently cited as proof of the inferiority of DCB vs DES, showed equal results for DCB and DES in the SVD and bifurcation subgroups [16]. The recently published ANDROMEDA patient level meta-analysis on DCB vs DES in SVD including a total of 1154 patients and 1360 lesions reported a lower risk of MACE at three years for DCB compared with DES, due to a lower risk of myocardial infarction and target vessel revascularization [7]. Other de novo indications for DCB are supported by randomized data from patients at high bleeding risk [6, 17, 18], acute coronary syndrome [19–21], or diabetes mellitus [22, 23].

The role of dissections after angioplasty is a subject of various controversial debates. The basic concern is that any dissection that is not treated with a stent may lead to vessel closure. This is precisely the point addressed by the ‘DCB only’ concept focusing on lesion preparation and, depending on its result, the decision between DCB and DES for local drug delivery is made [24, 25]. The algorithm is the basis for the combined use of DCB and DES. The lesions with potentially flow-limiting dissections or high-grade elastic recoil are treated with DES. The DCB treated lesions benefit from restoration of vasomotion [26] and an improvement of the lumen over time (late lumen enlargement) [27]. In addition, there is evidence of local regression of atherosclerosis [28, 29] with reduced inflammatory activity [30]. Systemic secondary prevention has still access to an uncaged artery. It has been shown that after ‘DCB only’ treatment, fibrous cap thickening and macrophage infiltration reduction were significantly greater in high-intensity statin therapy compared to moderate-intensity statin treatment [31]. Important to note, the rate of occluded arteries or the risk for myocardial infarction has been reported to be lower with ‘DCB only’ compared to DES [8, 32, 33].

The majority of interventional cardiologists is used to the old AHA classification to classify dissections. Here it was known that type A and B dissections had a low risk of vascular occlusion, while type C and higher were associated with higher event rates [34]. The question arises whether this classification is still valid for today’s strategies of lesion preparation and platelet inhibition. Angiographically, the assessment of flow and the dominance of the true lumen seems to be a guarantee for an open artery. In addition, physiological measurements and intravascular imaging (IVI) are integrated in our daily work. Specialty balloons allow for modification of dissections and thus further improve the primary angioplasty result [35]. The adequate sizing of both the lesion preparation devices and the DCB plays a central role here. IVI-guided lesion preparation leads to better initial lumen gain with reduced late lumen loss [36]. The creation of controlled dissections up to the media improves the angiographic and clinical outcome [37]. At least for paclitaxel DCB there is a new axiom of angioplasty: ‘the more you gain, the more you get’ [38].

**Fig. 1:**
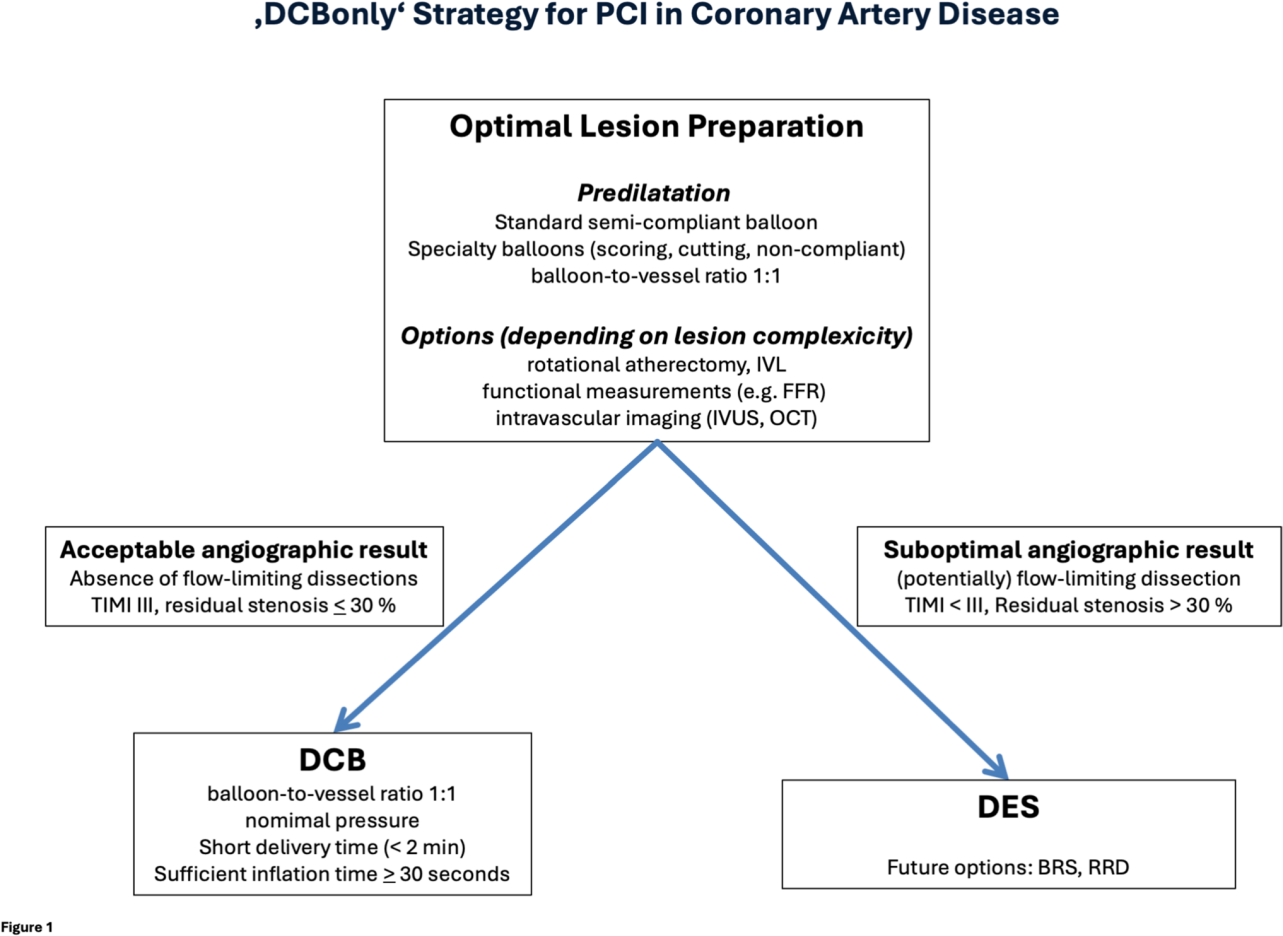
‘DCBonly’ strategy for PCI in coronary artery disease. PCI percutaneous coronary intervention, IVL intravascular lithoplasty, FFR fractional flow reserve, IVUS intravascular ultrasound, OCT optical coherence tomography, DCB drug coated balloon, DES drug eluting stent, BRS bioresorbable scaffold, RRD resorbable repair device. Updated from [25]

In the coming months and years, we expect the results of large clinical endpoint studies that will define the role of DCB in the treatment of coronary artery disease (e.g. SELUTION DeNovo, REVERSE, EBC DCB, PREVAIL GLOBAL IDE, BASKET B-ALL, etc.). Let’s be clear: The aim is not to replace DES but to redefine the specific use in clinical practice (Table 1). DCB therapy offers unique advantages and possibilities. The combination of DCB and DES, perhaps also with bioresorbable stents or resorbable repair devices, may improve long-term results in the future [39] (Fig. 1). DCB therapy has been in use for over 20 years, and it appears to be here to stay.

**Table 1:** What is the answer to the question “quo vadis”? Strengths and limitations of drug coated balloons (DCB) and drug eluting stents (DES) in the treatment of coronary artery disease. SVD small vessel disease. + means advantage, − means disadvantage for the respective treatment

	DES	DCB
Treatment duration	− Forever (permanent implant)	+ Seconds to minutes
Initial lumen gain	+	−
Late lumen enlargement	−	+ [27]
Controlled local drug release	+	Depending on coating technology [40]
Covering flow limiting dissections	+ [41]	−
Vasomotion	− caged vessel [26]	+ [26, 42]
Local access of systemic secondary prevention	− caged vessel	+
Atherosclerosis	− progression of neo-atherosclerosis [43]	+ Local regression of atherosclerosis [28–30]
Dual Antiplatelet Therapy	longer duration	Short duration, single antiplatelet therapy in high bleeding risk [6]
Suboptimal lesion preparation	+	− Cross-over to DES [25]
Device-associated events	− 2–3% per year [13, 14]	+ Flattening of the event curve in SVD [7]
Reducing number and length of permanent implants	−	+ [39]
Efficacy (target lesion revascularization)	+ [44]	− Dependent on lesion preparation [45]
Death, myocardial infarction	− [44]	+ lower for DCB in DES-ISR treatment [44]
